# Treatment-Related Serious Adverse Events of Immune Checkpoint Inhibitors in Clinical Trials: A Systematic Review

**DOI:** 10.3389/fonc.2021.621639

**Published:** 2021-05-11

**Authors:** Tao Ouyang, Yanyan Cao, Xuefeng Kan, Lei Chen, Yanqiao Ren, Tao Sun, Liangliang Yan, Bin Xiong, Bin Liang, Chuansheng Zheng

**Affiliations:** ^1^ Department of Radiology, Union Hospital, Tongji Medical College, Huazhong University of Science and Technology, Wuhan, China; ^2^ Hubei Province Key Laboratory of Molecular Imaging, Wuhan, China

**Keywords:** immune checkpoint inhibitors, treatment-related adverse events, treatment-related death, immune-related death, immune-related adverse events

## Abstract

**Background:**

Immune Checkpoint Inhibitors (ICI) have been progressively used in cancer treatment and produced unique toxicity profiles. This systematic review aims to comprehend the patterns and occurrence of treatment-related adverse events (trAEs) based on ICI.

**Methods:**

PICOS/PRISMA methods were used to identify published English-language on PubMed, Web of Science, and Scopus from 2015 to 2020. Published clinical trials on ICI monotherapy, combined ICIs, and ICI plus other treatment with tabulated data on grade≥3 trAEs were included. Odds ratio (OR), χ^2^ tests were used to analyze for effect size and associations.

**Results:**

This review included 145 clinical trials involving 21786 patients. Grade 3-5 trAEs were more common with ICI when they were plused with other treatments compared with ICI monotherapy(54.3% versus 17.7%, 46.1%, *p*<0.05). Grade 3-5 trAEs were also more common with CTLA-4 mAbs compared with anti-PD-1 and anti-PD-L1 (34.2% versus 15.1%, 13.6%, *p*<0.05). Hyperthyroidism (OR 3.8, 95%CI 1.7–8.6), nausea (OR 3.7, 95%CI 2.5–5.3), diarrhea (OR 2.7, 95%CI 2.2–3.2), colitis (OR 3.4, 95%CI 2.7–4.3), ALT increase (OR 4.9, 95%CI 3.9–6.1), AST increase (OR 3.8, 95%CI 3.0–4.9), pruritus (OR 2.4, 95%CI 1.5–3.9), rash (OR 2.8, 95%CI 2.1–3.8), fatigue (OR 2.8, 95%CI 2.2–3.7), decreased appetite (OR 2.4, 95%CI 1.5–3.8), and hypophysitis (OR 2.0, 95%CI 1.2–3.3) were more frequent with combined ICIs. Diarrhea (OR 8.1, 95%CI 6.4–10.3), colitis (OR 12.2, 95%CI 8.7–17.1), ALT increase (OR 5.1, 95%CI 3.5–7.4), AST increase (OR 4.2, 95%CI 2.8–6.3), pruritus (OR 4.1, 95%CI 2.0–8.4), rash (OR 4.4, 95%CI 2.9–6.8), hypophysitis (OR 12.1, 95%CI 6.3–23.4) were more common with CTLA-4 mAbs; whereas pneumonitis (OR 4.7, 95% CI 2.1–10.3) were more frequent with PD-1 mAbs.

**Conclusions:**

Different immune checkpoint inhibitors are associated with different treatment-related adverse events profiles. A comprehensive data in this systematic review will provide comprehensive information for clinicians.

## Introduction

Immune checkpoint inhibitors (ICI) are more and more being applied in many advanced solid cancers (1). ICI comprises cytotoxic T lymphocyte-associated protein-4 (CTLA-4), programmed cell death protein-1, and ligand-1 (PD-1 and PD-L1) monoclonal antibodies. They interrupt the negative regulation of T-cell responses and reactivate T-cell mediated antitumor immunity by interacting with receptors on dendritic cells(CTLA-4 receptors), T cells(PD-1 receptors), antigen-presenting cells (anti-PD-L1), or tumor cells (anti-PD-L1) (2). To enhance treatment effect, combination therapies that involve CTLA-4 plus PD-1/PD-L1 antagonist, and ICI with chemotherapy or antiangiogenic agents. The combination of targeted molecules and other immune-based therapies was more effective than monotherapy in some advanced cancers (3). With increasingly frequent use of ICI across different patterns, understanding their treatment-related Adverse Events is crucial. Combination use of ICIs also results in a higher risk of treatment-related adverse events (trAEs) compared with ICI monotherapy, including thyroid dysfunction, colitis, pneumonitis, dermatitis, and hepatitis (4, 5).

To date, toxicity data for immune checkpoint inhibitors are mainly available from randomized controlled trials. Evidence on the relative risk of toxicities especially grade≥3 AEs between different classes of agents remains limited. Therefore, we conducted a systematic review of monotherapy and combination therapy of ICIs in randomized controlled trials which specifically examined for differences in grade≥3 trAEs, immune-related adverse events (irAEs), and toxic death.

## Methods

### Eligibility Criteria

The following PICOS (Participants, Interventions, Control, Outcome and Study Design) criteria were used to define inclusion criteria for literature search:

1. Participants: The participants in the studies selected were patients with advanced cancer.
2. Interventions and control: The patients treated with different ICI monotherapy and combination.
3. Outcome: The occurrence of grade≥3 adverse events (AEs) in ICI monotherapy and combination.
4. Study design: The search criteria were conducted to identify published clinical trials of ICI monotherapy and combination which reported grade≥3 adverse events (AEs).

### Search Strategy and Study Selection

We performed a systematic search to recognize published relevant clinical trials of monotherapy and combination therapy of ICIs that reported trAEs. PubMed, Web of Science, and Scopus were searched for relevant literature using keywords “nivolumab”, “pembrolizumab”, “atezolizumab”, “avelumab”, “durvalumab”, “ipilimumab”, “tremelimumab”, “immune checkpoint inhibitors”, “PD-1 inhibitor”, “PD-L1 inhibitor”, and “CTLA-4 inhibitor”. The search was conducted from January 1, 2015 to March 1, 2020. Studies that met the following criteria were included: (1) clinical trials of advanced cancer treatment; (2) patients were treated with ICI monotherapy, CTLA-4 plus PD-1/PD-L1, or ICI plus other treatments; (3) reported tabulated data on immune-related or treatment-related grade≥3 adverse events; and (4) published in English. The literature search, research selection, and data extraction were acted separately by two reviewers (T.OY. and Y.Y.C), and any study that was thought to be potentially relevant was retrieved in full. Disagreements were resolved by consensus, but none occurred.

### Data Extraction

The ICI agent, author name and year, phase, tumor type, number of patients, number of treatment-related 3-5 toxicities, number of deaths, death reason were collected from each selected study. trAEs, treatment-related deaths, irAEs and immune-related deaths were collected and analyzed. Adverse events that were not described as treatment-related or possibly unrelated to treatment were excluded. The data was extracted from the main text and **Supplementary Information**.

### Statistical Analysis

Baseline characteristics were summarized using frequency and percentage. For most common trAEs, percentages were reported and used in all analyses. Odds ratio (OR) with 95% confidence intervals (CI) were used to calculate the results of different ICI drugs and the use of ICI patterns (monotherapy, combined therapy and ICI plus chemotherapy). The *P*-values were calculated using the χ^2^ test and was listed in the tables. *P*-value <0.05 was considered statistically significant. All analyses were performed by SPSS 25.0.

## Results

### Study Selection and Characteristics

**Figure 1:**
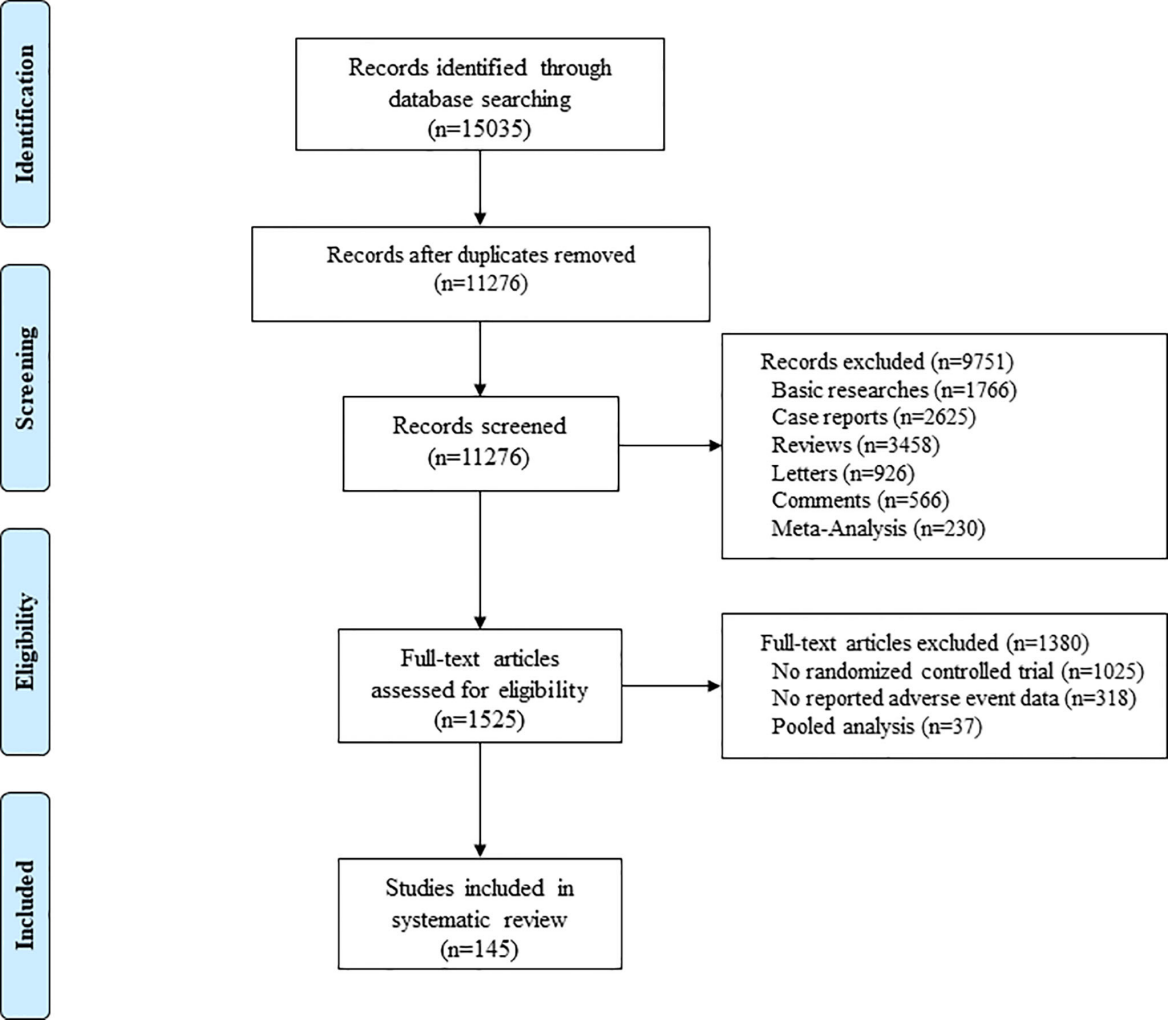
Flow Diagram of the Study Selection Process.

The initial database originally identified 15035 relevant clinical trials. Finally, 145 studies involving 21786 patients meeting the inclusion criteria were included into this systematic review (**Figure 1**) (6–146). **Additional Table S1** summarizes the trial and patient characteristics. The trials involved the treatment as monotherapy (n=104), combined therapy of two ICIs (n=20), and ICI in combination with chemotherapy or other treatments (n=33). The ICI monotherapy arms that were used in our study included PD-1 inhibitors (n=75), PD-L1 inhibitors (n=18), and CTLA-4 inhibitors (n=17). The combined ICIs treatment arm that was used in our study included PD-1 inhibitors combining with CTLA-4 inhibitors (n=18), and PD-L1 inhibitors plus CTLA-4 inhibitors (n=2). Moreover, the included clinical trials involved the cancer type of melanoma (n=37), lung cancer (n=36), renal cell cancer (n=10), urothelial cancer (n=9), gastrointestinal cancer (n=7), head and neck squamous cell cancer (n=7), breast cancer (n=7), and other cancers (n=32). Almost all trials had tumor metastatic, except for 17 trials. This systematic review included 48 phase I, 8 phase I/II, 59 phase II and 30 phase III clinical trials.

For the systematic review, we analyzed adverse events that reported by more than 5% of the clinical trials. We defined ICI monotherapy, combined therapy of two ICIs, and ICI plus other treatments as cohort 1, cohort 2 and cohort 3, respectively.

### Overall Incidence of Grade≥3 trAEs

Collectively, all of the included clinical trials reported over 100 kinds of adverse events. On the whole, at least 1 grade 3 or higher adverse event occurred in 5,599 (25.7%) of 21,786 patients in 145 studies. In the ICI monotherapy treatment group, at least 1 grade 3 or higher adverse event occurred in 2889 (17.7%) of 16395 patients in 104 studies. Moreover, in the combined ICIs treatment group, 1531 (46.1%) of 3321 patients from 20 studies developed grade≥3 AEs, and 1179 (54.3%) of 2170 in ICI plus other treatments group. The most commonly reported trAEs were hypothyroidism, nausea, hyperthyroidism, diarrhea, colitis, alanine aminotransferase (ALT) increase, Aspartate aminotransferase (AST) increase, rash, arthralgia, myalgia, pruritus, pneumonitis, fatigue, decreased appetite, and hypophysitis. The incidence of treatment-related grade 3-5 AEs, treatment-related death, immune-related grade 3-5 AEs, and immune-related death are shown in **Table 1**. **Additional Table S2** reports the percentages of these common adverse events in three cohorts and different ICI agents.

**Table 1:** Overall incidence of grade≥3 trAEs and irAEs.

Cohort/agent	No. of patients	Treatment-related 3-5 AEs	Treatment-related death	Immune-related 3-5 AEs	Immune-related death
1	**16295**	**2889 (17.7%)**	**71 (0.4%)**	**969 (5.9%)**	**34 (0.2%)**
2	**3321**	**1531 (46.1%)**	**39 (1.2%)**	**814 (24.5%)**	**16 (0.5%)**
3	**2170**	**1179 (54.3%)**	**58 (2.7%)**	**230 (10.6%)**	**5 (0.2%)**
PD-1 inhibitors	**10407**	**1567 (15.1%)**	**46 (0.4%)**	**336 (3.2%)**	**17 (0.2%)**
PD-L1 inhibitors	**3350**	**455 (13.6%)**	**14 (0.4%)**	**81 (2.4%)**	**8 (0.2%)**
CTLA-4 inhibitors	**2538**	**867 (34.2%)**	**11 (0.4%)**	**552 (21.7%)**	**1 (0.04%)**

Cohort 1: ICI monotherapy; Cohort 2: combined therapy of two ICIs; Cohort 3: ICI plus other treatments.

The incidence of AEs in different types of ICI included only monotherapy trials.

### Incidence of Grade≥3 trAEs According to Study Groups

Grade≥3 trAEs were more frequent in the group of ICI plus other treatments (cohort 3) compared with the groups of ICI monotherapy (cohort1) and combined therapy of two ICIs (cohort2) (54.3, 95% CI 52.2-56.4; 17.7, 95% CI 17.1-18.3; 46.1, 95% CI 44.4-47.8, respectively). However, Grade≥3 irAEs were more common in cohort 2 compared with that of in cohort1 and cohort 3 (24.5, 95% CI 23.0-26.0; 5.9, 95% CI 5.6-6.3; 10.6, 95% CI 9.3-11.9), these results are provided in **Table 2**. Grade≥3 hyperthyroidism (OR 3.8, 95% CI 1.7–8.6), nausea (OR 3.7, 95% CI 2.5–5.3), diarrhea (OR 2.7, 95% CI 2.2–3.2), colitis (OR 3.7, 95% CI 2.7–4.3), ALT increase (OR 4.9, 95% CI 3.9–6.1), AST increase (OR 3.8, 95% CI 3.0–4.9), pruritus (OR 2.4, 95% CI 1.5–3.9), rash (OR 2.8, 95% CI 2.1–3.8), fatigue (OR 2.8, 95% CI 2.2–3.7), decreased appetite (OR 2.4, 95% CI 1.5–3.8), and hypophysitis (OR 2.0, 95% CI 1.2–3.3) were more frequent with cohort 2 compared with cohort 1 (**Table 3**).

**Table 2:** Comparison of grade ≥3 trAEs and irAEs from 3 cohorts.

	Cohort 1 *Vs* Cohort 2	*P* value	Cohort 1 *Vs* Cohort 3	*P* value	Cohort 2 Vs Cohort 3	*P* value
**Treatment-related 3-5 AEs**	17.7 (17.1-18.3) *Vs* 46.1 (44.4-47.8)	*P*<0.001	17.7 (17.1-18.3) *Vs* 54.3 (52.2-56.4)	*P*<0.001	46.1 (44.4-47.8) *Vs* 54.3 (52.2-56.4)	*P*<0.001
**Treatment-related death**	0.4 (0.3-0.5) *Vs* 1.2 (0.8-1.5)	*P*<0.001	0.4 (0.3-0.5) *Vs* 2.7 (2.0-3.4)	*P*<0.001	1.2 (0.8-1.5) *Vs* 2.7 (2.0-3.4)	*P*<0.001
**Immune-related 3-5 AEs**	5.9 (5.6-6.3) *Vs* 24.5 (23.0-26.0)	*P*<0.001	5.9 (5.6-6.3) *Vs* 10.6 (9.3-11.9)	*P*<0.001	24.5 (23.0-26.0) *Vs* 10.6 (9.3-11.9)	*P*<0.001
**Immune-related death**	0.2 (0.1-0.3) *Vs* 0.5 (0.2-0.7)	*P*=0.004	0.2 (0.1-0.3) *Vs* 0.2 (0-0.4)	*P*=0.836	0.5 (0.2-0.7) *Vs* 0.2 (0-0.4)	*P*=0.14

Cohort 1: ICI monotherapy; Cohort 2: combined therapy of two ICIs; Cohort 3: ICI plus other treatments.

**Table 3:** Comparison of grade ≥3 trAEs between cohort 1 and 2.

	OR (95% CI)	*P* value
**Hyperthyroidism**	3.8 (1.7-8.6)	*P*=0.001
**Nausea**	3.7 (2.5-5.3)	*P*<0.001
**Diarrhea**	2.7 (2.2-3.2)	*P*<0.001
**Colitis**	3.4 (2.7-4.3)	*P*<0.001
**ALT increase**	4.9 (3.9-6.1)	*P*<0.001
**AST increase**	3.8 (3.0-4.9)	*P*<0.001
**Pruritus**	2.4 (1.5-3.9)	*P*<0.001
**Rash**	2.8 (2.1-3.8)	*P*<0.001
**Fatigue**	2.8 (2.2-3.7)	*P*<0.001
**Decreased appetite**	2.4 (1.5-3.8)	*P*<0.001
**Hypophysitis**	2.0 (1.2-3.3)	*P*=0.007

Cohort 1: ICI monotherapy; Cohort 2: combined therapy of two ICIs.

### Incidence of Grade ≥3 trAEs According to ICI Class

Grade ≥3 trAEs were more likely to happen in CTLA-4 compared with PD-1 ICI and PD-L1 ICI (34.2, 95% CI 32.3-36.0; 15.1, 95% CI 14.4-15.7; 13.6, 95% CI 12.4-14.7). Similar results were obtained in Grade≥3 irAEs. In comparison of irAEs from the monotherapy showed that CTLA-4 had a significantly higher frequency of Grade≥3 irAEs compared with PD-1 and PD-L1 (21.7, 95% CI 20.1-23.4,; 3.2, 95% CI 2.9-3.6, *p* < 0.001; 2.4, 95% CI 1.9-2.9, *p* < 0.001) (**Table 4**). The differences of AEs in different types of ICI included only monotherapy trials. Diarrhea (OR 8.1, 95% CI 6.4–10.3), colitis (OR 12.2, 95% CI 8.7–17.1), ALT increase (OR 5.1, 95% CI 3.5–7.4), AST increase (OR 4.2, 95% CI 2.8–6.3), pruritus (OR 4.1, 95% CI 2.0–8.4), rash (OR 4.4, 95% CI 2.9–6.8), hypophysitis (OR 12.1, 95% CI 6.3–23.4) were more frequent when patients received the treatment of CTLA-4 ICI; whereas pneumonitis (OR 4.7, 95% CI 2.1–10.3) were more common when patients received the treatment of PD-1 monoclonal antibodies (**Table 5**).

**Table 4:** Comparison of grade ≥3 trAEs and irAEs from 3 ICIs.

	PD-1 *Vs* PD-L1	*P* value	PD-1 *Vs* CTLA-4	*P* value	PD-L1 *Vs* CTLA-4	*P* value
**Treatment-related 3-5 AEs**	15.1 (14.4-15.7) *Vs* 13.6 (12.4-14.7)	*P*=0.036	15.1 (14.4-15.7) *Vs* 34.2 (32.3-36.0)	*P*<0.001	13.6 (12.4-14.7) *Vs* 34.2 (32.3-36.0)	*P*<0.001
**Treatment-related death**	0.4 (0.3-0.6) *Vs* 0.4 (0.2-0.6)	*P*=0.854	0.4 (0.3-0.6) *Vs* 0.4 (0.2-0.7)	*P*=0.953	0.4 (0.2-0.6) *Vs* 0.4 (0.2-0.7)	*P*=0.928
**Immune-related 3-5 AEs**	3.2 (2.9-3.6) *Vs* 2.4 (1.9-2.9)	*P*=0.017	3.2 (2.9-3.6) *Vs* 21.7 (20.1-23.4)	*P*<0.001	2.4 (1.9-2.9) *Vs* 21.7 (20.1-23.4)	*P*<0.001
**Immune-related death**	0.2 (0.1-0.2) *Vs* 0.2 (0.1-0.4)	*P*=0.372	0.2 (0.1-0.2) *Vs* 0 (0-0.1)	*P*=0.133	0.2 (0.1-0.4) *Vs* 0 (0-0.1)	*P*=0.052

The incidence of AEs in different types of ICI included only monotherapy trials.

**Table 5:** Comparison of grade ≥3 trAEs between CTLA-4 and PD1/PD-L1.

	PD1/PD-L1 OR (95% CI)	*P* value	CTLA-4 OR (95% CI)	*P* value
**Diarrhea**			8.1 (6.4-10.3)	*P*<0.001
**Colitis**			12.2 (8.7-17.1)	*P*<0.001
**ALT increase**			5.1 (3.5-7.4)	*P*<0.001
**AST increase**			4.2 (2.8-6.3)	*P*<0.001
**Pruritus**			4.1 (2.0-8.4)	*P*<0.001
**Rash**			4.4 (2.9-6.8)	*P*<0.001
**Pneumonitis**	4.7 (2.1-10.3)	*P*<0.001		
**Hypophysitis**			12.1 (6.3-23.4)	*P*<0.001

### Incidence of Treatment-Related Death and Immune-Related Death

Among the studies, 59 clinical trials reported treatment-related death, and a total of 165 such deaths reported (**Additional Table S1**). The occurrence of treatment-related death in all studies was 0.75% (165 of 21786). The incidence of treatment-related death was more common with ICI plus chemotherapy (cohort 3) compared with ICI monotherapy (cohort 1) (2.7, 95% CI 2.0-3.4 versus 0.4, 95% CI 0.3-0.5). Immune-related death were more frequent in cohort 2 compared with cohort 1 (0.5, 95% CI 0.2-0.7 versus 0.2, 95% CI 0.1-0.3). According to the ICI class, the comparison of treatment-related death and immune-related death were not statistically significant. All the results were provided in **Tables 2** and **4**.

## Discussion

We performed a systematic review of monotherapy, combination therapy of ICIs, and ICI inhibitor with other treatment relevant adverse events based on data from published clinical trials. Meanwhile, we executed a subgroup analysis to compare the difference of PD-1, PD-L1 and CTLA-4 inhibitors in the treatment-related and immune-related adverse events. This systematic review analyzed the number of each adverse event associated with different ICIs to draw precise statistical inferences that were close or even the same as the results of individual-level data, which was different from the meta-analyses that used continuous summary statistics based on the large-sample theory. To date, this systematic review was the first and most comprehensive study of treatment-related serious adverse events for three treatment modalities involving ICIs. A comprehensive analysis of the unique adverse events associated with different ICIs reported in clinical trials will aid clinicians in providing comprehensive information.

The precise mechanism of trAEs is still unknown, it may relate to block inhibitory checkpoints and activate T-cell immune. Recently, several hypotheses are suggested. First, the use of immune checkpoint inhibitors disrupts the immunologic homeostasis and reduces T-cell tolerance (147). Second, there is some cross-reaction of T-cells between tumor cells and normal tissue (148). Third, immune checkpoint inhibitors can increase scales of preexisting autoantibodies and inflammatory cytokines (149). For these facts, activated T-cells assault normal tissue resulting in trAEs. Although many clinical practice guidelines of trAEs have been published recently, such as the American Society of Clinical Oncology and National Comprehensive Cancer Network guidelines (150), trAEs present an entirely new set of clinical challenges. These autoimmune toxicities are incredibly diverse, potentially affecting almost every organ system (151). In our study, the most common reported trAEs were diarrhea, colitis, ALT increase, AST increase, and fatigue. Less common but potentially more serious trAEs include pneumonitis and hypophysitis. Less common still are dreaded effects on the heart and central nervous system, which were not mentioned in our article.

Our study has demonstrated that the incidence of serious trAEs associated with PD-1/PD-L1 agents was significantly lower than that of the CTLA-4 inhibitors (15.1%, 13.6% and 34.2%, respectively). In general, anti-CTLA-4 agents was more toxic than anti-PD-1/PD-L1 agent, which is consistent with our results (152). However, there was no significant difference in the treatment-related death among PD-1, PD-L1, and CTLA-4 inhibitors (0.4%, 0.4% and 0.4%, respectively). This finding may be explained that the majority of serious AEs (grade≥3) were reversible after the systemic apply of glucocorticoids, then, manage properly. CTLA-4 and PD-1 are separate pathways from both a spatial and a chronological standpoint (153). CTLA-4 can prevent the interaction between class II major histocompatibility complex molecules of the antigen presenting cells, and the T-cell receptor to inhibit the activation of T cells (154). More recently, CTLA-4 has also been implicated in the function of Tregs, which suppress effector T-cell activation and function (155, 156). However, the precise mechanism and role for CTLA-4 Treg function is a much-debated topic. Anti-CTLA-4 antibodies can lead to an interruption of peripheral tolerance by activated T-cell and Treg cells (157). To date, the precise mechanism of PD-1 in T-cell tolerance is not completely clear. It plays its function mostly in peripheral tissues, inducing a homeostatic inhibition of previously activated T-cells (158). Besides, PD-1 may inhibit T-cell function and survival directly, by blocking early activation signals that are promoted by CD28, or indirectly through IL-2 (159).

This review indicated that the majority of reported serious adverse events were related to immune-related, including pneumonitis, diarrhea, colitis, elevated ALT and AST, hyperthyroidism, and hypophysitis. High-dose corticosteroids were the first line for treating irAEs and may help to enable their proper management. If not detected and cured early, these immune-related toxicities will make progression and may even endanger life. This systematic review showed that the immune- related serious adverse events was significantly higher in CTLA-4 inhibitors than that of in PD-1 and PD-L1 inhibitors(21.7%, 3.2%, 2.4% respectively). According to the previous studies, the incidence of grade 3 or higher irAEs ranges from 15% to 42% in anti-CTLA-4 agents, 5% to 10% in anti-PD-1 agents, and 1% to 7% in anti-PD-L1 agents (150). Our results are in agreement with these previous research. Nonetheless, there was no statistical difference in immune-related deaths among the three monotherapies.

According to the previous study reported, one of the most common adverse events caused by ICI was endocrinological diseases (160). Although all the endocrine glands may be invaded, in our study, the thyroid and hypophysis were the most constantly invaded organs. Although not fully understood, the mechanism of immune-related thyroid dysfunction comprises autoimmune thyroiditis, mediated by T-cell cytotoxicity, natural killer cells, and PD-1/PD-L1 expression in thyroid tissue (161). Hypophysitis is more likely to occur in patients with anti-CTLA-4 agents as an on-target effect of ectopic CTLA-4 protein expression in the pituitary gland, antibody-dependent cell-mediated cytotoxicity (ADCC), and activation of the complement pathway (162). This conclusion is consistent with our research that the prevalence of hypophysitis in patients receiving anti-CTLA-4 agents was higher than that of patients receiving anti-PD-1/PD-L1 agents (odds ratio [OR] 12.1, 95% confidence interval [CI] 6.3–23.4). Skin toxicity (rash and pruritus), digestive tract disorders (Diarrhea, Colitis, ALT increase and AST increase), and rheumatologic disorders(Arthralgia/Myalgia) are the most frequent adverse events occurred in patients with anti-CTLA-4 agents, except for pneumonitis which is common reported in anti-PD-1/anti-PD-L1 agents(4.7, 95% CI 2.1-10.3). Although the precise underlying mechanism remains unclear, there was a hypothesis indicated that alveolar macrophages probably hyperactivated in patients who received anti-PD-1 agents. This hypothesis is sustained by the phenomenon that interstitial macrophages and alveolar cells express repulsive guidance molecule B (RGMB) in the surface, which may act as a ligand to PD-L2 (163).

Indeed, ICI monotherapy benefits just a few patients, with objective response rates (ORR) of about 15-25%, and even lower for pancreatic carcinoma, prostate cancer, ovarian carcinoma, triple-negative breast cancer, and microsatellite stable colorectal cancer. It may be attributed to tumor heterogeneity and tumor resistance (164). Therefore, many researches were focused on seeking the combination strategies which can improve anti-tumor immunity and increase treatment efficacy. To date, the majority of combination strategy was immune checkpoint inhibitors with antiangiogenic agents, ICI with chemotherapy, and combined of two ICIs. Some previous literatures had demonstrated that a higher incidence of adverse events was recorded in employing ICI combination compared with ICI monotherapy (3, 4). Interestingly, our research indicated that ICI plus chemotherapy or antiangiogenic agent(cohort 3) had the highest adverse events and treatment-related death. The higher frequency of severe AEs in cohort 3 probably illustrated by the multiple targets of TKIs. The toxicities include not only adverse events that were related to the barricade of the VEGR/VEGFR pathway, but also adverse events that were caused by extra targets inhibition (165). Generally, the combination therapy strategy had a high occurrence of trAEs, including any grade trAEs, and grade 3 or higher trAEs. In contrast, ICI combination can increase the benefit derived from ICI monotherapy in tumors which already responsive to ICI monotherapy. However, due to the lack of enough clinical results comparing the ICI combination to ICI monotherapy, it is currently difficult to conduct accurate risk-benefit analysis of ICI combination.

Pneumonitis and cardiac causes were the most common treatment-related deaths in our study. According to reports, the incidence of ICI-related pneumonitis that was reported in clinical trials in ICI monotherapy is 2.5%–5.0%, and in combination therapy ranges from 7% to 10% (166). Moreover, compared with CTLA-4 inhibitors, PD-1/PD-L1 agents performed a higher incidence and severity of pulmonary adverse events (167). This finding is consistent with our results. The treatment of pneumonitis is ICI cessation, systemic steroids, and immunosuppressive medications. If ICIs are reused, ICI-related pneumonitis can recrudesce in 20% of patients (168). The most common ICI-related cardiotoxicity is myocarditis. ICI-related cardiotoxicity is relatively limited but life-threatening and deadly. Therefore, clinicians should more focus on cardiotoxicity, especially of grade 3 or higher in immunotherapy (169).

There are limitations in our study. The heterogeneity among included studies cannot be ignored. In addition, cancer types, phase of trial, number of patients and criteria for reporting adverse events are the source of heterogeneity. Moreover, given the differences in the number of patients included in subgroups analyzed, bias was inevitable to some extent.

In conclusion, this systematic review demonstrated that the combination treatment of ICIs is associated with a significantly higher occurrence of serious adverse events compared to ICI monotherapy. Meanwhile, our results also indicated that anti-CTLA-4 agents have a higher incidence of serious adverse events compared with anti-PD-1/anti-PD-L1 agents. Pneumonitis and cardiac toxicity were the main causes of treatment-related death, but the incidence of treatment-related deaths was low.

## Data Availability Statement

The original contributions presented in the study are included in the article/**Supplementary Material**. Further inquiries can be directed to the corresponding authors.

## Author Contributions

CZ, BL, and BX contributed conception/design. TO, YC, and XK contributed in collection of assembly of data. LC, YR, TS, and LY performed data analysis and interpretation. All authors contributed to the article and approved the submitted version.

## Funding

This study was supported by a grant from the National Natural Science Foundation of China (Nos. 81873919).

## Conflict of Interest

The authors declare that the research was conducted in the absence of any commercial or financial relationships that could be construed as a potential conflict of interest.
