# Supplementary material for: Treatment-Related Serious Adverse Events of Immune Checkpoint Inhibitors in Clinical Trials: A Systematic Review
Source: Front Oncol. 2021 May 11;11:621639. doi: 10.3389/fonc.2021.621639 (PMC8144509; doi:10.3389/fonc.2021.621639)
Supplement: Supplementary file 1 [file DataSheet_1.pdf]

Additional Table S1. Characteristics of included trials.

<table>
<tr><th></th><th>Author, year</th><th>Phase</th><th>Cancer type</th><th>Stage</th><th>Intervention</th><th>NO. of patients</th><th>Fatal adverse events</th></tr>
<tr><td>1</td><td>Hiroyuki, 2020</td><td>III</td><td>urothelial cancer</td><td>metastatic</td><td>pembrolizumab</td><td>30</td><td>0</td></tr>
<tr><td>2</td><td>Shin, 2020</td><td>II</td><td>ovarian cancer</td><td>metastatic</td><td>pembrolizumab</td><td>21</td><td>0</td></tr>
<tr><td>3</td><td>Neal, 2019</td><td>I/ II</td><td>lung cancer</td><td>metastatic</td><td>nivolumab</td><td>109</td><td>1</td></tr>
<tr><td rowspan="3">4</td><td rowspan="3">Lillian, 2019</td><td rowspan="3">II</td><td rowspan="3">head and neck squamous cell carcinoma</td><td rowspan="3">metastatic</td><td>durvalumab</td><td>65</td><td>0</td></tr>
<tr><td>tremelimumab</td><td>65</td><td>0</td></tr>
<tr><td>durvalumab+ tremelimumab</td><td>133</td><td>1</td></tr>
<tr><td rowspan="2">5</td><td rowspan="2">Yung, 2019</td><td rowspan="2">II</td><td rowspan="2">gastric/gastroesophageal junction adenocarcinoma</td><td rowspan="2">metastatic</td><td>pembrolizumab</td><td>31</td><td>0</td></tr>
<tr><td>pembrolizumab + chemotherapy</td><td>25</td><td>0</td></tr>
<tr><td>6</td><td>Leisha, 2019</td><td>I</td><td>triple-negative breast cancer</td><td>metastatic</td><td>atezolizumab</td><td>116</td><td>0</td></tr>
<tr><td>7</td><td>Ken, 2019</td><td>III</td><td>gastric/gastroesophageal junction adenocarcinoma</td><td>metastatic</td><td>nivolumab</td><td>152</td><td>0</td></tr>
<tr><td>8</td><td>Adams, 2019</td><td>II</td><td>triple-negative breast cancer</td><td>metastatic</td><td>pembrolizumab</td><td>170</td><td>0</td></tr>
<tr><td>9</td><td>Paolo, 2019</td><td>III</td><td>melanoma</td><td>metastatic</td><td>nivolumab</td><td>206</td><td>0</td></tr>
<tr><td>10</td><td>Yutaka, 2019</td><td>I</td><td>gastric/gastroesophageal junction adenocarcinoma</td><td>metastatic</td><td>durvalumab</td><td>22</td><td>0</td></tr>
<tr><td>11</td><td>Manish, 2019</td><td>II</td><td>esophageal cancer</td><td>metastatic</td><td>pembrolizumab</td><td>121</td><td>1</td></tr>
<tr><td>12</td><td>Stephen, 2019</td><td>II</td><td>B-cell lymphoma</td><td>non-metastatic</td><td>nivolumab</td><td>121</td><td>0</td></tr>
<tr><td>13</td><td>Makoto, 2019</td><td>I</td><td>lung cancer</td><td>metastatic</td><td>pembrolizumab</td><td>38</td><td>1</td></tr>
<tr><td>14</td><td>Robert, 2019</td><td>II</td><td>hodgkin lymphoma</td><td>non-metastatic</td><td>pembrolizumab</td><td>210</td><td>0</td></tr>
<tr><td>15</td><td>Janice, 2019</td><td>I</td><td>thyroid cancer</td><td>metastatic</td><td>pembrolizumab</td><td>22</td><td>0</td></tr>
<tr><td>16</td><td>Adams, 2019</td><td>II</td><td>triple-negative breast cancer</td><td>metastatic</td><td>pembrolizumab</td><td>84</td><td>0</td></tr>
<tr><td>17</td><td>Kenji, 2019</td><td>II</td><td>uterine cervical cancer</td><td>metastatic</td><td>nivolumab</td><td>64</td><td>0</td></tr>
<tr><td>18</td><td>Paul, 2019</td><td>II</td><td>merkel cell carcinoma</td><td>metastatic</td><td>pembrolizumab</td><td>50</td><td>1</td></tr>
</table>

19	Ahmad, 2019	II	melanoma	metastatic	ipilimumab	44	0
					ipilimumab+ interferon-α2b	39	0
20	Melissa, 2019	I	melanoma	metastatic	pembrolizumab	40	0
21	Ulrich, 2019	I	melanoma	metastatic	avelumab	51	0
22	Hellmann, 2018	III	lung cancer	metastatic	nivolumab	391	2
					nivolumab+ ipilimumab	576	7
23	Yelena, 2018	I/ II	esophagogastric cancer	metastatic	nivolumab	59	0
					nivolumab+ ipilimumab	101	1
24	Sandra, 2018	II	sarcoma	metastatic	nivolumab	42	0
					nivolumab+ ipilimumab	42	6
25	Alexand, 2018	III	melanoma	metastatic	pembrolizumab	509	1
26	David, 2018	II	renal cell carcinoma	metastatic	atezolizumab	103	0
27	Robert, 2018	III	head and neck squamous cell carcinoma	metastatic	nivolumab	236	2
28	Antonio, 2018	I	glioblastoma	non-metastatic	nivolumab	10	0
					nivolumab+ ipilimumab	30	2
29	Matteo, 2018	III	melanoma	metastatic	pembrolizumab	368	1
					ipilimumab	168	0
30	Migden, 2018	II	cutaneous squamous-cell carcinoma	metastatic	cemiplimab	59	3
31	Charles, 2018	II	gastric/gastroesophageal junction adenocarcinoma	metastatic	pembrolizumab	259	0
32	Brigette, 2018	II	nasopharyngeal carcinoma	metastatic	nivolumab	45	1
33	Ranee, 2018	I	head and neck squamous cell carcinoma	metastatic	pembrolizumab	192	0
34	Richard, 2018	II	melanoma	metastatic	ipilimumab	6	0
35	James, 2018	III	melanoma	metastatic	nivolumab	268	0
36	Daniel, 2018	I	urothelial cancer	metastatic	atezolizumab	95	0
37	Toyoaki, 2018	III	lung cancer	metastatic	atezolizumab	56	0

38	David, 2018	II	lung cancer	metastatic	atezolizumab	137	1
39	Makoto, 2018	I	head and neck squamous cell carcinoma	metastatic	pembrolizumab	26	0
40	Colevas, 2018	I	head and neck squamous cell carcinoma	metastatic	atezolizumab	32	0
41	Dan, 2018	II	lung cancer	metastatic	durvalumab	112	0
42	Adra, 2018	II	germ-cell tumors	metastatic	pembrolizumab	12	0
43	Hope, 2018	I	breast cancer	metastatic	pembrolizumab	25	0
44	Jong, 2018	II	lung cancer	metastatic	nivolumab	100	1
45	Weber, 2017	III	melanoma	metastatic	nivolumab	452	0
					ipilimumab	453	2
46	Arjun, 2017	II	urothelial carcinoma	metastatic	atezolizumab	119	1
47	Achim, 2017	III	lung cancer	metastatic	atezolizumab	609	0
48	Antonia, 2017	III	lung cancer	non-metastatic	durvalumab	475	7
49	Thomas, 2017	I/ II	urothelial carcinoma	metastatic	durvalumab	191	2
50	Van, 2017	II	anal canal carcinoma	metastatic	nivolumab	37	0
51	Michael, 2017	II	colorectal cancer	metastatic	nivolumab	74	0
52	Wolchok, 2017	III	melanoma	metastatic	nivolumab	313	1
					ipilimumab	311	1
					nivolumab+ ipilimumab	313	2
53	Bellmunt, 2017	III	urothelial carcinoma	metastatic	pembrolizumab	266	4
54	Jason, 2017	II	melanoma	metastatic	ipilimumab	95	0
55	Necchi, 2017	II	urothelial carcinoma	metastatic	atezolizumab	137	0
56	Solange, 2017	II	lung cancer	metastatic	atezolizumab	659	1
57	Wei, 2017	II	lymphocytic leukemia	non-metastatic	pembrolizumab	25	0

58	Bert, 2017	I	colorectal carcinoma	metastatic	pembrolizumab	23	0
59	Hui, 2017	I	lung cancer	metastatic	pembrolizumab	101	0
60	Toyoaki, 2017	II	lung cancer	metastatic	nivolumab	35	1
61	Ott, 2017	I	anal carcinoma	metastatic	pembrolizumab	25	0
62	Joshua, 2017	II	head and neck squamous cell carcinoma	metastatic	pembrolizumab	171	1
63	Pier, 2017	I	B-cell lymphoma	non-metastatic	pembrolizumab	18	0
64	Yoshihiko, 2017	III	renal cell carcinoma	non-metastatic	nivolumab	37	0
65	Eytan, 2017	II	uterine leiomyosarcoma	metastatic	nivolumab	12	0
66	Noboru, 2017	I	lung cancer	metastatic	nivolumab	17	0
67	Yung, 2017	II	gastric/gastroesophageal junction adenocarcinoma	metastatic	ipilimumab	57	0
68	Naoya, 2017	I	melanoma	metastatic	pembrolizumab	42	2
69	Keun, 2017	I	lung cancer	metastatic	nivolumab	18	0
70	Naoya, 2017	II	melanoma	metastatic	nivolumab	23	0
71	Tara, 2017	II	melanoma	metastatic	pembrolizumab	947	0
72	Dai, 2017	II	hodgkin lymphoma	non-metastatic	nivolumab	17	0
73	Martin, 2016	III	lung cancer	metastatic	pembrolizumab	154	1
74	Ferris, 2016	III	head and neck squamous cell carcinoma	non-metastatic	nivolumab	236	2
75	Stephen, 2016	II	melanoma	metastatic	ipilimumab	46	0
					nivolumab+ ipilimumab	94	3
76	Rita, 2016	I	triple-negative breast cancer	metastatic	pembrolizumab	32	1
77	Jonathan, 2016	II	urothelial carcinoma	metastatic	atezolizumab	310	0
78	Eggermont 2016	II	melanoma	metastatic	ipilimumab	471	5

79	Christophe 2016	I/ II	bladder cancer	metastatic	durvalumab	61	0
80	Alexander 2016	I	hodgkin lymphoma	non-metastatic	nivolumab	81	1
81	Scott, 2016	I	lung cancer	metastatic	nivolumab	52	0
82	Anas, 2016	II	hodgkin lymphoma	non-metastatic	nivolumab	80	1
83	Paul, 2016	II	merkel-cell carcinoma	metastatic	pembrolizumab	26	0
84	Melinda, 2016	I	melanoma	metastatic	ipilimumab	33	0
85	Padmanee, 2016	I/ II	urothelial carcinoma	metastatic	nivolumab	78	1
86	Philippe, 2016	I	hodgkin lymphoma	non-metastatic	pembrolizumab	31	0
87	Toshio, 2016	I	lung cancer	metastatic	pembrolizumab	10	0
88	James, 2015	III	melanoma	metastatic	nivolumab	313	1
					ipilimumab	311	1
					nivolumab+ ipilimumab	313	0
89	Le, 2015	II	colorectal carcinomas	metastatic	pembrolizumab	41	0
90	Hossein, 2015	III	lung cancer	metastatic	nivolumab	287	1
91	Robert, 2015	III	renal cell carcinoma	metastatic	nivolumab	406	0
92	Julie, 2015	III	lung cancer	metastatic	nivolumab	131	0
93	Caroline, 2015	III	melanoma	metastatic	nivolumab	206	0
94	Edward, 2015	I	lung cancer	metastatic	pembrolizumab	495	1
95	Caroline, 2015	III	melanoma	metastatic	pembrolizumab	550	0
					ipilimumab	256	1
96	Stephen, 2015	I	hodgkin lymphoma	non-metastatic	nivolumab	23	0
97	Michael, 2015	I	melanoma	metastatic	ipilimumab	46	0
					nivolumab+ ipilimumab	94	0
98	Naiyer, 2015	II	lung cancer	metastatic	nivolumab	117	2

99	Robert, 2015	II	renal cell carcinoma	metastatic	nivolumab	167	0
100	Scott, 2015	I	lung cancer	metastatic	nivolumab	129	3
101	David, 2015	I	renal cell carcinoma	metastatic	nivolumab	34	0
102	Lisa, 2015	II	melanoma	metastatic	ipilimumab	103	0
103	Disa, 2015	II	melanoma	metastatic	ipilimumab	53	1
104	Yamazaki, 2015	II	melanoma	metastatic	ipilimumab	20	0
105	Yoshihiko, 2020	III	renal cell carcinoma	non-metastatic	nivolumab+ ipilimumab	38	1
106	Celeste, 2019	III	melanoma	metastatic	nivolumab+ ipilimumab	358	1
107	Michael, 2019	II	melanoma	metastatic	nivolumab+ ipilimumab	124	0
108	Motzer, 2018	III	renal cell carcinoma	metastatic	nivolumab+ ipilimumab	547	8
109	Hussein, 2018	II	melanoma	metastatic	nivolumab+ ipilimumab	94	1
110	Kenjiro, 2018	II	melanoma	metastatic	nivolumab+ ipilimumab	30	0
111	Michael, 2018	I	melanoma	metastatic	ipilimumab+ pembrolizumab	22	0
112	Margaret, 2018	I	melanoma	metastatic	nivolumab+ ipilimumab	94	1
113	Matthew, 2017	I	lung cancer	metastatic	nivolumab+ ipilimumab	77	0
114	Scott, 2016	I	lung cancer	metastatic	durvalumab+ tremelimumab	103	3
115	Jeffrey, 2016	II	melanoma	metastatic	nivolumab+ ipilimumab	138	0
116	Alex, 2020	I/ II	B-cell lymphoma	non-metastatic	durvalumab+ ibrutinib	61	0
117	Erminia, 2019	II	oropharyngeal cancer	non-metastatic	nivolumab+ vaccine	24	0
118	Boku, 2019	II	gastric/gastroesophageal junction adenocarcinoma	metastatic	nivolumab+ chemotherapy	21	0
					nivolumab+ chemotherapy	18	0

119	Sylvia, 2019	I	triple-negative breast cancer	metastatic	Atezolizumab+ nab-Paclitaxel	33	0
120	James, 2019	I/ II	lung cancer	metastatic	pembrolizumab + erlotinib	12	0
					pembrolizumab + gefitinib	7	0
121	Hiroji, 2019	III	triple-negative breast cancer	metastatic	Atezolizumab+ nab-Paclitaxel	34	0
122	Socinski, 2018	III	lung cancer	metastatic	atezolizumab+ bevacizumab	393	11
123	David, 2018	II	renal cell carcinoma	metastatic	atezolizumab+ bevacizumab	101	1
124	Paz-Ares, 2018	III	lung cancer	metastatic	pembrolizumab + chemotherapy	278	10
125	Gandhi, 2018	III	lung cancer	metastatic	pembrolizumab + chemotherapy	405	27
126	Michael, 2018	I	melanoma	metastatic	pembrolizumab + PEG-IFN	17	0
127	Asim, 2018	I	renal cell carcinoma	metastatic	nivolumab+ sunitinib	33	0
					nivolumab+ pazopanib	20	0
128	Michael, 2018	I	renal cell carcinoma	metastatic	pembrolizumab + axitinib	52	0
129	Alex, 2018	I/ II	hodgkin lymphoma	non-metastatic	nivolumab+ brentuximab	61	0
130	Scott, 2018	I	lung cancer	metastatic	nivolumab+ erlotinib	21	0
131	Richard, 2018	II	melanoma	metastatic	ipilimumab+ ATRA	4	0
132	John, 2018	I	lung cancer	metastatic	nivolumab+ interleukin	21	1
133	Hendrik, 2018	I	biliary tract cancer	metastatic	ramucirumab+ pembrolizumab	26	0
134	Mamta, 2018	I	urothelial cancer	metastatic	pembrolizumab + docetaxel	12	0
135	Jason, 2017	II	lung cancer	metastatic	ipilimumab+ talimogene	95	0
136	Anthony, 2017	I	lung cancer	metastatic	pembrolizumab + utomilumab	23	0
137	Chad, 2017	I	lung cancer	metastatic	ipilimumab+ radiation	35	0

138	Sandra, 2017	I	gastrointestinal stromal tumor	metastatic	ipilimumab+ dasatinib	24	0
139	Matthew, 2017	I	melanoma	metastatic	ipilimumab+ imatinib	35	0
140	Corey, 2016	II	lung cancer	metastatic	pembrolizumab + chemotherapy	59	1
141	Naiyer, 2016	I	lung cancer	metastatic	nivolumab+ chemotherapy	56	0
142	Igor, 2016	I	melanoma	metastatic	ipilimumab+ T-VEC	19	0
143	Edurne, 2016	II	lung cancer	metastatic	ipilimumab+ chemotherapy	39	5
144	Yamazaki, 2015	II	melanoma	metastatic	ipilimumab+ dacarbazine	15	0
145	Geoffrey, 2015	II	melanoma	metastatic	nivolumab+ vaccine	33	0

Additional Table S2. Percentages of most common TRAEs

	Cohort1<br>n=16295	Cohort2<br>n=3321	Cohort3<br>n=2170	PD-1<br>n=10407	PD-L1<br>n=3350	CTLA-4<br>n=2538
**Hypothyroidism**	0.3	0.3	0.4	0.3	0.5	0.3
**Hyperthyroidism**	0.1	0.3	0.1	0.0	0.2	0.1
**Nausea**	0.4	1.5	2.6	0.2	0.8	0.5
**Diarrhea**	2.1	5.4	4.9	1.0	1.2	7.8
**Colitis**	1.2	4.0	1.6	0.5	0.4	5.2
**ALT increase**	1.0	4.5	3.1	0.5	1.3	2.4
**AST increase**	0.9	3.3	3.0	0.4	1.6	1.8
**Arthralgia**	0.2	0.1	0.5	0.1	0.4	0.3
**Myalgia**	0.1	0.0	0.2	0.0	0.1	0.0
**Pruritus**	0.3	0.7	0.3	0.1	0.6	0.6
**Rash**	0.7	2.0	2.3	0.4	0.9	1.8
**Pneumonitis**	0.8	0.5	1.5	0.7	1.3	0.3
**Fatigue**	1.0	2.8	4.8	0.9	1.3	0.9
**Decreased appetite**	0.4	0.8	1.8	0.2	0.7	0.4
**Hypophysitis**	0.3	0.6	0.5	0.1	0.1	1.4

Cohort 1: ICI monotherapy; Cohort 2: combined therapy of two ICIs; Cohort 3: ICI plus other treatments.
